# Feasibility study of an automated Strabismus screening Test using Augmented Reality and Eye-tracking (STARE)

**DOI:** 10.1038/s41433-023-02566-0

**Published:** 2023-05-04

**Authors:** Nisha Nixon, Peter B. M. Thomas, Pete R. Jones

**Affiliations:** 1 Department of Ophthalmology, Norfolk and Norwich University Hospitals NHS Foundation Trust, Colney Lane, Norwich, NR4 7UY UK; 2 NIHR Biomedical Research Centre for Ophthalmology, Moorfields Eye Hospital NHS Foundation Trust and UCL Institute of Ophthalmology, London, EC1V 9EL UK; 3 Department of Optometry and Visual Sciences, School of Health & Psychological Sciences, City, University of London, London, EC1V 0HB UK

**Keywords:** Ocular motility disorders, Physical examination

## Abstract

**Background:**

New digital technologies (augmented reality headsets, eye-tracking) may potentially allow for automated assessments of ocular misalignment. Here, we evaluate the feasibility of a novel, open-source strabismus test (“STARE”) as an automated screening tool.

**Methods:**

Work progressed in 2 phases. In phase 1 (“development”), we used Fresnel prisms to elicit horizontal misalignments of known magnitude (1–40 prism dioptres) in orthotropic controls. In phase 2 (“validation”), we applied the system to adults with an established diagnosis of strabismus, and quantified the ability of the test to distinguish between those with horizontal misalignment and those without. Agreement between the alternate prism cover test measurements and STARE measurements was computed using Bland–Altman plots and product-moment correlation coefficients.

**Results:**

Seven orthotropic controls and nineteen patients with strabismus were recruited (mean age 58.7 ± 22.4 years). STARE was able to identify the presence of horizontal strabismus with an area under the curve of 1.00 (100% sensitivity and 100% specificity). The mean difference (bias) {95% CI} was 2.1 {−1.8, 9.9} prism dioptres, and the 95% coefficient of repeatability {95% CI} was ±27.9 {14.8, 50.8} prism dioptres. The Pearson correlation between APCT and STARE was r_24_ = 0.62, *P* < 0.001.

**Conclusions:**

STARE shows promise as a simple, automated tool for performing a screening assessment of strabismus. It is a rapid (60 s) test that can be performed using a consumer augmented reality headset with integrated eye-tracking, and might conceivably be used remotely by non-specialists in future as a means of highlighting individuals needing face-to-face specialist care.

## Introduction

The United Kingdom National Screening Committee (UKNSC) recommends orthoptist-led screening for ‘visual defects’ at 4-5 years of age, including ‘amblyopia, refractive error and strabismus’ [1]. Currently, assessment of strabismus requires a trained examiner [2], adept at using prisms [3], in order to obtain an accurate diagnosis of an ocular deviation. These face-to-face assessments are nuanced and detailed, and could not be readily replaced by automated technologies. However, digital screening tools might provide a helpful “triage” function: augmenting existing services by highlighting individuals in need of specialist orthoptic assessment, and helping to target overstretched resources [4]. To that end, here we introduce STARE (Strabismus screening Test using Augmented Reality and Eye-tracking): a free open-source, test designed to mimic the alternate prism cover test using “off the shelf” hardware, and which could conceivably be administered by a non-specialist outside of a standard clinical environment.

Eye-tracking has already been used previously (for example, in combination with laptops [5, 6], video goggles [7], and virtual reality headsets [8, 9]) to objectively quantify ocular misalignment in prism dioptres. Eye-trackers offer many advantages, including the ability to monitor eye movement even under occlusion, and to quantify deviation of the eyes beyond the fixed increments afforded by prisms in clinical use. Further, using an eye-tracker mounted within an enclosed, head-mounted display (as in, a virtual reality headset) obviates the need for the patient’s head to be fixed in place, and provides complete control of ambient illumination and the targets presented to the patient.

The novelty of the headset-based strabismus test described in the present work is two-fold. Firstly, the work is non-profit and open-source (under a permissive MIT license), allowing interested parties to scrutinise, adapt, or extend its inner workings without restriction (https://github.com/petejonze/STARE). Secondly, previous studies that have used virtual reality headsets to assess ocular misalignment [8–10] have used artificial, simplistic stimuli (single targets on a uniform black background). In the present study we instead allow the patient to view their surroundings as they are using stereoscopic pass-through cameras i.e., ‘augmented reality’ [11], allowing the user to see the world around them, rather than completely replacing it, as in ‘virtual reality’ [12]. A young child, for instance, would still be able to see their parent or the clinician during the test. From a validation perspective, the use of pass-through cameras also enabled us to ‘simulate’ ocular misalignment in orthotropic controls, by placing Fresnel prisms over the eyepieces of the headset – thereby providing a simple and unambiguous way of quantifying the sensitivity of the system. From a technical perspective, augmented reality also makes the software extremely simple and lightweight. Indeed, since there is no complex virtual environment to code, and since we used ‘off-the-shelf’ eye-tracking and camera technology (see Methods), the basecode for the entire data collection pipeline is only around 100 lines long, thus making it easy to distribute and providing minimal scope for error.

The present study was intended to provide a preliminary evaluation of feasibility, and proceeded in two phases. In phase 1 (“development”), we used Fresnel prisms to elicit horizontal misalignments of known magnitude (1–40 prism dioptres) in 7 orthotropic controls. By using prisms of varying strength, we were able to calibrate the system to determine how changes in gaze position in millimetres, mapped to rotation of the eye, in equivalent prism dioptres. We were also able to determine the smallest detectable misalignment, as an indication of the sensitivity of the system. In phase 2 (“validation”), we further applied the system to 19 adults with established diagnosis of strabismus, and quantified the ability (sensitivity/specificity) of the test to distinguish between those with horizontal misalignment and those without (orthotropic controls and patients with only vertical deviations).

## Methods

### Participants

Participants were 19 adults with ocular misalignment and 7 orthotropic controls.

Patients were opportunistically sampled from Oculomotility Clinic at Norfolk and Norwich University Hospitals NHS Foundation Trust between February 2022 and June 2022. The only inclusion criterion was a diagnosed disturbance of oculomotility. People aged under 18 or unable to perform the alternate prism cover test due to poor visual acuity were excluded. The orthotropic controls were staff at the Eye clinic. The study was approved by an NHS Research Ethics Committee (19/NE/0305), and carried out in accordance with the tenets of the Declaration of Helsinki. Written informed consent was obtained from all patients prior to testing.

### Reference measure

Each patient recruited to the study underwent full orthoptic assessment, including alternate prism cover testing at 6 m distance fixation. The orthoptists performing the former were masked to the results of the STARE test, which was performed subsequently on the same day in a different room.

### The novel ‘STARE’ (Strabismus screening Test using Augmented Reality and Eye-tracking) procedure

**Fig. 1:**
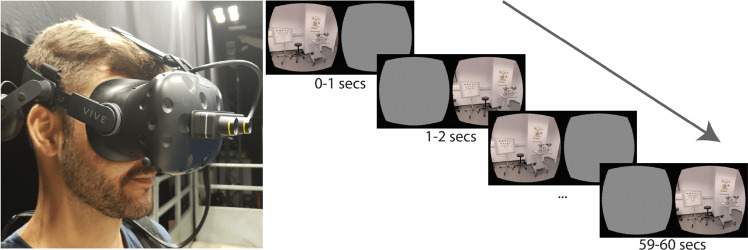
Augmented reality headset worn by participants, and schematic illustrating the alternate occlusion of each eye during the 60-s test. See Fig. 2 for example data trace.

The STARE (Strabismus screening Test using Augmented Reality and Eye-tracking) procedure required each participant to wear the Tobii Pro HTC Vive virtual reality headset with integrated eye-tracking (HTC Corporation, Taoyuan, Taiwan). Participants wore their habitual optical (distance) correction, as required, and during the test were asked to fixate a target 6 m away in the room. After initial binocular viewing, each eye was alternately occluded for 1 s (Fig. 1) for the duration of the test (60 s), whilst eye position was recorded continuously by the eye-tracker integrated within the headset (accuracy 0.5°–1.1° and sampling frequency of 120 Hz [13]. An attached computer displayed both the patient’s view and their gaze position during the test, so an operator was able to monitor performance in real time. The computer program was written in Unity 3D, and is available online (https://github.com/petejonze/STARE), along with the associated Matlab analysis code (see next).

**Fig. 2:**
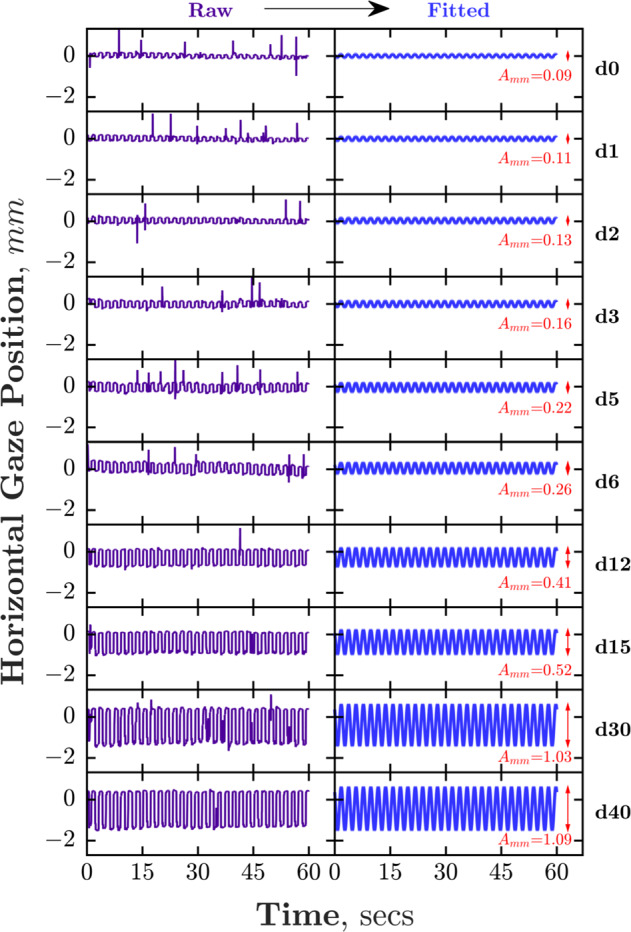
Horizontal gaze position for a single eye recorded by STARE in a control participant. Gaze position is plotted over time in a control participant with simulated horizontal strabismus of varying magnitudes (d0 = 0 prism dioptres).

Analysis of eye position data was performed in Matlab, and involved fitting of a sine wave to the raw gaze data for each eye, as shown in Fig. 2. The period of the sine wave was fixed at two seconds, given the one-second alternate occlusion of the eyes, and the magnitude was a free parameter, thus allowed to vary depending on size of deviation.

### Phase 1: Technical development & preliminary validation in controls

In order to establish the relationship between gaze position, as measured in millimetres by the Tobii eye tracker, and deviation of the eyes measured in prism dioptres, a validation protocol was performed on seven control participants. These control participants underwent multiple runs of STARE, each with a different strength of base-out Fresnel prism (0, 1, 2, 3, 5, 6, 12, 15, 30, 40 prism dioptres for each participant) placed over the right eyepiece in the virtual reality headset, inducing a horizontal strabismus of known magnitude. The runs were performed in succession on the same day, and eye position was recorded as described above. Figure 2 illustrates the raw gaze position data of one such participant, upon which sine waves were fitted.

### Phase 2: Assessing the ability (sensitivity/specificity) of STARE to detect horizontal deviations in patients and controls

Agreement between the alternate prism cover test measurements and STARE measurements was computed using Bland–Altman plots and product-moment correlation coefficients. The performance of STARE in identifying presence of horizontal strabismus was assessed using its diagnostic parameters, including accuracy, sensitivity, specificity and area under the receiver operating curve (AUROC). In general, *P* values <0.05 were considered statistically significant, and non-parametric bootstrapping (*N* = 20,000) was used to generate 95% confidence intervals.

## Results

### Phase 1: Technical development & preliminary validation in controls

**Fig. 3:**
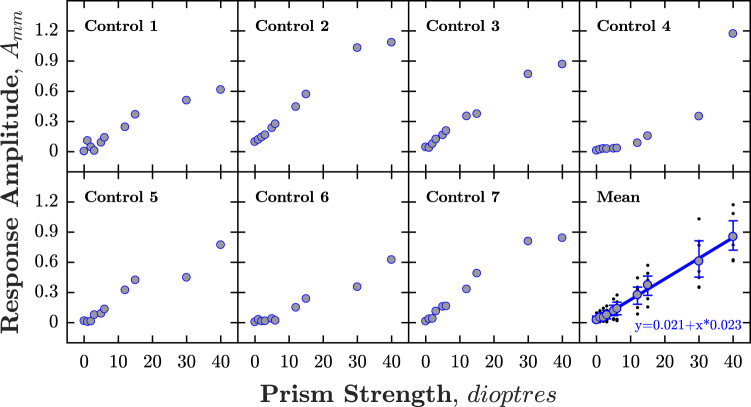
Response amplitude plotted against strength of simulated horizontal deviation in each control participant. Mean response amplitude of all control participants is indicated at bottom right, along with the fitted calibration function (used in Phase 2 to map response amplitude, in mm, to horizontal misalignment, in equivalent prism dioptres).

In all control participants, the amplitude of horizontal re-fixation movement in millimetres increased as the strength of simulated horizontal deviation increased (Fig. 3). The mean amplitude of the horizontal re-fixation movement was plotted against strength of simulated deviation, and the relationship fitted in order to estimate prism dioptre deviations in patients undergoing the STARE procedure.

### Phase 2: Assessing the ability (sensitivity/specificity) of STARE to detect horizontal deviations in patients and controls

Nineteen patients were recruited, with a mean age of 58.7 ± 22.4 years. They included 8 patients with an esodeviation, 7 with an exodeviation, and 10 with a vertical deviation (2 of whom had no accompanying horizontal deviation).

**Fig. 4:**
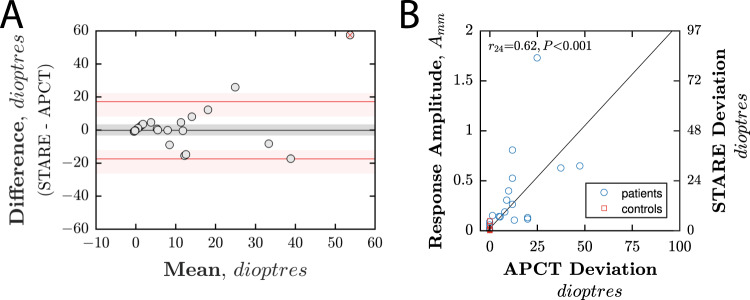
Agreement between measurements of horizontal deviation made using the standard alternate prism cover test (APCT), performed by an orthoptist, and the novel STARE method. **A** Bland–Altman plot showing mean agreement (black line), and 95% limits of agreement (red lines), both with ±95% confidence intervals. Note that the top right point was not included when computing the limits of agreement shown, but statistics with and without this point included are given in the main text. **B** Scatter plot, including unity line and Pearson correlation statistics.

Figure 4 shows Bland–Altman and scatter plots comparing measurements of horizontal deviation, in prism dioptres, made using the standard alternate prism cover test (APCT) method, and the novel STARE method. The mean difference (bias) {95% CI} was 2.1 {−1.8, 9.9} prism dioptres, and the 95% coefficient of repeatability {95%CI} was ±27.9 {14.8, 50.8} prism dioptres, decreasing to ±17.3 {12.0, 26.4} prism dioptres when, as shown in Fig. 4A, one outlying point was removed. As shown in Fig. 4B, the Pearson correlation between APCT and STARE was r_24_ = 0.62, *P* < 0.001.

**Fig. 5:**
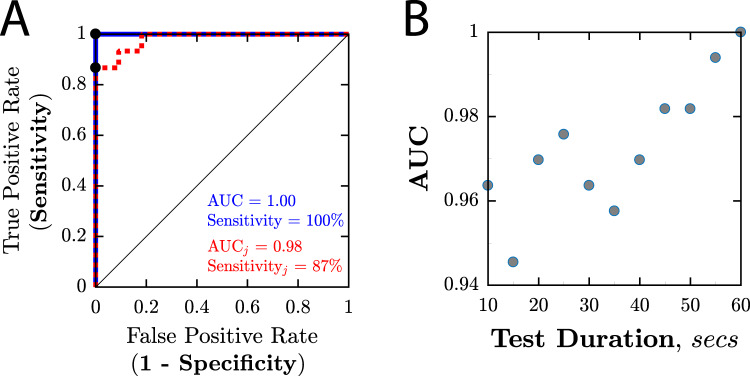
Ability of STARE to identify (i.e., screen for) patients with horizontal strabismus. **A** Mean receiver operating characteristics (ROCs). The blue line corresponds to the original raw data shown previously in Fig. 4B. The red line shows the mean ROC from simulations in which a realistic amount of random noise (“jitter”) was introduced into the STARE measurements (see body text for details). **B** AUC values as a function of test duration (i.e., when deviation values were computed only using the first *N* seconds of eye-tracking data; see Fig. 2 for example raw data traces).

As shown in Fig. 5 (blue line), the ability of STARE to identify presence of horizontal strabismus, irrespective of magnitude, appeared high, with an Area Under the Curve (AUC) of 1.00 (i.e., 100% sensitivity, 100% specificity). However, close inspection of Fig. 4B suggests that this result may be misleadingly optimistic, with only a very marginal separation (in terms of STARE scores) between some individuals with/without true horizontal deviations. Therefore, to provide a more nuanced estimate of sensitivity and specificity, we simulated additional ROC curves after introducing a small degree of noise into the data (by slightly jittering each STARE estimated deviation value by a random amount, drawn from a Gaussian distribution with standard deviation equal to the standard deviation observed between the 7 healthy controls). When this was done (Fig. 5A, red dashed line), the mean AUC reduced to 0.98, with a sensitivity of 87% when specificity was fixed at 100% (Fig. 4: red dashed line).

To establish the necessary duration of the STARE protocol, and to determine in particular whether the same screening accuracy could be achieved with a shorter test, the ROC analysis described previously (and illustrated graphically in Fig. 5A, blue line), was repeated, using only the first *N* seconds of eye-tracking data. The main finding is given in Fig. 5B, which shows that AUC values improved progressively as more data were used, not reaching an asymptote before the full 60 s. This indicates that a test duration shorter than 60 s would diminish the accuracy of the test substantively, and implies that a test duration longer than 60 s might be beneficial.

## Discussion

This study describes a novel, automated method for screening of strabismus (“Strabismus screening Test using Augmented Reality and Eye-tracking”: STARE). STARE combines free, open source software with a commercially available headset with integrated eye-tracking (HTC Vive Pro Eye – though several other similar headsets are also available) to perform an automated alternate cover test. The patients’ task was simply to fixate a physical target in the Eye clinic for 60 s.

It was encouraging to note that STARE was able to detect moderate and severe cases of horizontal strabismus with reasonable (87%) sensitivity and 100% specificity. Given our intended use of STARE as a screening tool, its 100% specificity is particularly important – indeed an estimated prevalence of strabismus of 1.93% [14] means that a specificity lower than 98% would result in more false positive referrals than true positives. The test was reasonably quick (60 s once the patient was wearing the headset and instructed to look at a target) and tolerable (100% completion rate amongst control participants and patients), so might conceivably be used remotely by non-specialists in future as a means of highlighting those needing face-to-face specialist care.

We were keen to determine whether STARE, in its current iteration, could also quantify horizontal deviations, as would be expected in a specialist setting. Of note, our technical validation procedure based upon seven control participants with simulated deviations highlighted large variation in response amplitude, particularly at higher magnitude deviations. This individual variability may account for the wider limits of agreement in our Bland–Altman analysis compared to previous virtual reality tests (95% limit of agreement 27.9 prism dioptres, compared to 11.3 [8] and 3.1 [9]). One extreme outlier can be identified in our patient data – an 83 year-old myopic patient with an alternating esotropia measuring 25 prism dioptres on APCT and 82 prism dioptres on STARE. The incorporated eye trackers within the virtual reality headset rely on pupil tracking under infra-red illumination to determine eye movement in millimetres [13], and extremes of axial length or vertex distance would lead to errors in the conversion of this into a prism dioptre deviation. This may well explain the gross overestimation of deviation of our myopic patient mentioned above.

### Comparisons to previous research

Previous authors have attempted to automate strabismus assessment using photographs [15–17] and binocular OCT [18]. The former in particular has significant potential for large-scale strabismus screening, but fails to capture the nuances of the alternate prism cover test, including identification of phorias and abnormal retinal correspondence. The assessments are also limited by room lighting, image quality and spectacle correction.

Commercially available virtual reality headsets overcome many of these limitations, by offering complete control of targets and illumination, and are a natural choice of device to digitise the alternate prism cover test. Yeh et al. (2021) [8] compare the performance of a virtual reality headset-based test with the APCT in a cohort of 38 patients and show good agreement for both horizontal and vertical deviations. However, their test requires a skilled operator, able to assess presence of eye movement during the test and change target position accordingly. Instead, as in the present study, Miao et al. [9] report an automated virtual reality headset-based test, requiring a non-skilled operator. They find good agreement with the alternate prism cover test in the assessment of horizontal deviation in 17 patients. However, all patients were required to have their interpupillary distance and axial length measured in addition to performing the test, in order for an angle of deviation to be estimated.

The aforementioned studies used single fixation targets on a uniform black background, reported to be at 6 m distance within a ‘virtual environment’. Nevertheless, both note uncertainties around the patient’s perceived fixation distance, and indeed Yeh et al. (2021) [8] find a tendency to esotropia with their virtual reality test, suggesting accommodation and significant convergence to the target. In our study, instead of depriving the patient of their usual surroundings and distance cues, we allow the patient to view a faithful stereoscopic representation of the room they are in. There is thus complete flexibility over choice of fixation target – a young child could, for instance, be asked to look at their parent’s face for 60 s, whilst the STARE protocol is performed.

### Test limitations

In order for pupil tracking to be accurate, the virtual reality headset must be fitted correctly on the patient’s head. The headset used in this study would be too big for use in young children, but was selected as it is relatively inexpensive and readily commercially available. In future, we expect smaller and cheaper virtual reality headsets to become widespread. The eye trackers used in this study were unable to measure torsion, and STARE cannot be used in those with nystagmus. STARE requires the patient to be able to fixate on a target throughout the test, the optimum duration of which appears to be a full 60 s (Fig. 5B). The protocol used in the present study does not differentiate between manifest and latent deviations.

### Study limitations and future work

There are several limitations to this feasibility study of STARE. External validity is limited by our small sample of patients. Only horizontal deviations were considered in comparisons of size of deviation between APCT and STARE, and no intra- or inter-observer data was recorded. Ocular deviation was also only measured in primary position with STARE.

This study forms the preparatory work for future iterations of the test, where we aim to use computer-generated augmented reality overlays of fixation targets within our real-world environment, in order to eliminate the effect of head movement and more readily determine size of deviation for both near and distance. By introducing a cover-uncover test into the protocol, we aim to also differentiate manifest from latent deviations.

## Conclusions

In summary, this pilot study indicates STARE shows promise as a simple, automated tool for performing a screening assessment of strabismus. It is a rapid, tolerable test that can be performed using an unmodified, commercial-grade virtual reality headset. Although the data in this study were collected in a hospital eye clinic, such a test may in future be used by those unable to reach a traditional healthcare setting. Future work aims to identify and quantify both manifest and latent deviations, and to supplement the data from this real-world three-dimensional test using augmented reality overlays.

## Summary

### What was known before

- Previous authors have attempted to automate strabismus assessment using photographs and binocular OCT, but these fail to capture the nuances of the alternate prism cover test.
- Commercially available virtual reality headsets overcome many of these limitations, by offering complete control of targets and illumination, and are a natural choice of device to digitise the alternate prism cover test.

### What this study adds

- This study describes a novel, open-source, automated method for screening of strabismus (“Strabismus screening Test using Augmented Reality and Eye-tracking”: STARE).In our study, instead of depriving the patient of their usual surroundings and distance cues, we allow the patient to view a faithful stereoscopic representation of the room they are in, using augmented reality. STARE is a rapid, tolerable screening test of strabismus that may in future be used by those unable to reach a traditional healthcare setting.

## Data Availability

The data that support the findings of this study are not openly available due to reasons of sensitivity and are available from the corresponding author upon reasonable request.

## References

[1] United Kingdom National Screening Committee. Recommendation on vision defects screening in children. London: Public Health England; 2013. https://legacyscreening.phe.org.uk/vision-child.

[2] Hrynchak PK, Herriot C, Irving EL (2010). Comparison of alternate cover test reliability at near in non-strabismus between experienced and novice examiners. Ophthalmic Physiol Opt.

[3] Thompson JT, Guyton DL (1983). Ophthalmic prisms. Measurement errors and how to minimize them. Ophthalmology.

[4] Royal College of Ophthalmologists. RCOphth calls for action on workforce to reduce strain on ophthalmology services. 2021 https://www.rcophth.ac.uk/news-views/rcophth-calls-for-action-on-workforce-to-reduce-strain/.

[5] Chen ZH, Fu H, Lo WL, Chi Z, Xu B (2018). Eye-tracking-aided digital system for strabismus diagnosis. Healthc Technol Lett.

[6] Yehezkel O, Belkin M, Wygnanski-Jaffe T (2020). Automated diagnosis and measurement of strabismus in children. Am J Ophthalmol.

[7] Weber KP, Rappoport D, Dysli M, Schmückle Meier T, Marks GB, Bockisch CJ (2017). Strabismus measurements with novel video goggles. Ophthalmology.

[8] Yeh PH, Liu CH, Sun MH, Chi SC, Hwang YS (2021). To measure the amount of ocular deviation in strabismus patients with an eye-tracking virtual reality headset. BMC Ophthalmol.

[9] Miao Y, Jeon JY, Park G, Park SW, Heo H (2020). Virtual reality-based measurement of ocular deviation in strabismus. Comput Methods Programs Biomed.

[10] Nesaratnam N, Thomas P, Vivian A (2017). Stepping into the virtual unknown: feasibility study of a virtual reality-based test of ocular misalignment. Eye.

[11] Carmigniani J, Furht B. Augmented reality: an overview. In: Furht B editor. Handbook of augmented reality. 1st edn. New York: Springer; 2011. pp 3–46.

[12] Azuma RT (1997). A survey of augmented reality. Presence.

[13] High Tech Computer Corporation. HTC Vive Pro Eye. 2019. https://www.vive.com/uk/product/vive-pro-eye/overview/.

[14] Hashemi H, Pakzad R, Heydarian S, Yekta A, Aghamirsalim M, Shokrollahzadeh F (2019). Global and regional prevalence of strabismus: a comprehensive systematic review and meta-analysis. Strabismus.

[15] Tenório Albuquerque Madruga Mesquita MJ, Azevedo Valente TL, de Almeida JDS, Meireles Teixeira JA, Cord Medina FM, Dos (2021). A mhealth application for automated detection and diagnosis of strabismus. Int J Med Inform.

[16] Zheng C, Yao Q, Lu J, Xie X, Lin S, Wang Z (2021). Detection of referable horizontal strabismus in children’s primary gaze photographs using deep learning. Transl Vis Sci Technol.

[17] Huang X, Lee SJ, Kim CZ, Choi SH (2021). An automatic screening method for strabismus detection based on image processing. PLoS One.

[18] Chopra R, Mulholland PJ, Tailor VK, Anderson RS, Keane PA (2018). Use of a binocular optical coherence tomography system to evaluate strabismus in primary position. JAMA Ophthalmol.

